# Direct Common Carotid Artery Puncture for Endovascular Treatment of Acute Large Vessel Ischemic Stroke in a Patient With Congenital Rare Variant of the Great Vessels

**DOI:** 10.7759/cureus.11127

**Published:** 2020-10-24

**Authors:** Samuel Plant, Biraj M Patel

**Affiliations:** 1 Medicine, Virginia Tech Carilion School of Medicine, Roanoke, USA; 2 Neurointerventional Surgery, Carilion Clinic, Roanoke, USA

**Keywords:** ischemic stroke, interventional neuroradiology, mechanical embolectomy, direct carotid artery puncture, aortic arch, rare variant

## Abstract

This report describes a case of acute left middle cerebral artery ischemic stroke that occurred in a patient with unique anatomy of the vessels arising from the aortic arch that remained undiagnosed until the age of 36. The nature of the anatomical variance proved problematic in establishing access to the carotid artery via traditional transfemoral or transbrachial approaches, and direct access was established via left carotid puncture. The patient regained substantial neurologic function. The direct carotid approach described below serves as a viable alternate route to establishing reperfusion in patients with complex or problematic aortic arch anatomy.

## Introduction

Congenital variants and abnormalities of the aortic arch are associated with a wide spectrum of symptomology [1] as well as an increased risk of neurological events [2]. In this anonymized case report, the patient remained undiagnosed and asymptomatic until found down at his home. It was not known that the patient had any vascular anatomical variance until arch aortogram and several attempts to catheterize the origin of the left common carotid artery revealed the complex anatomical rare variant.

## Case presentation

**Figure 1:**
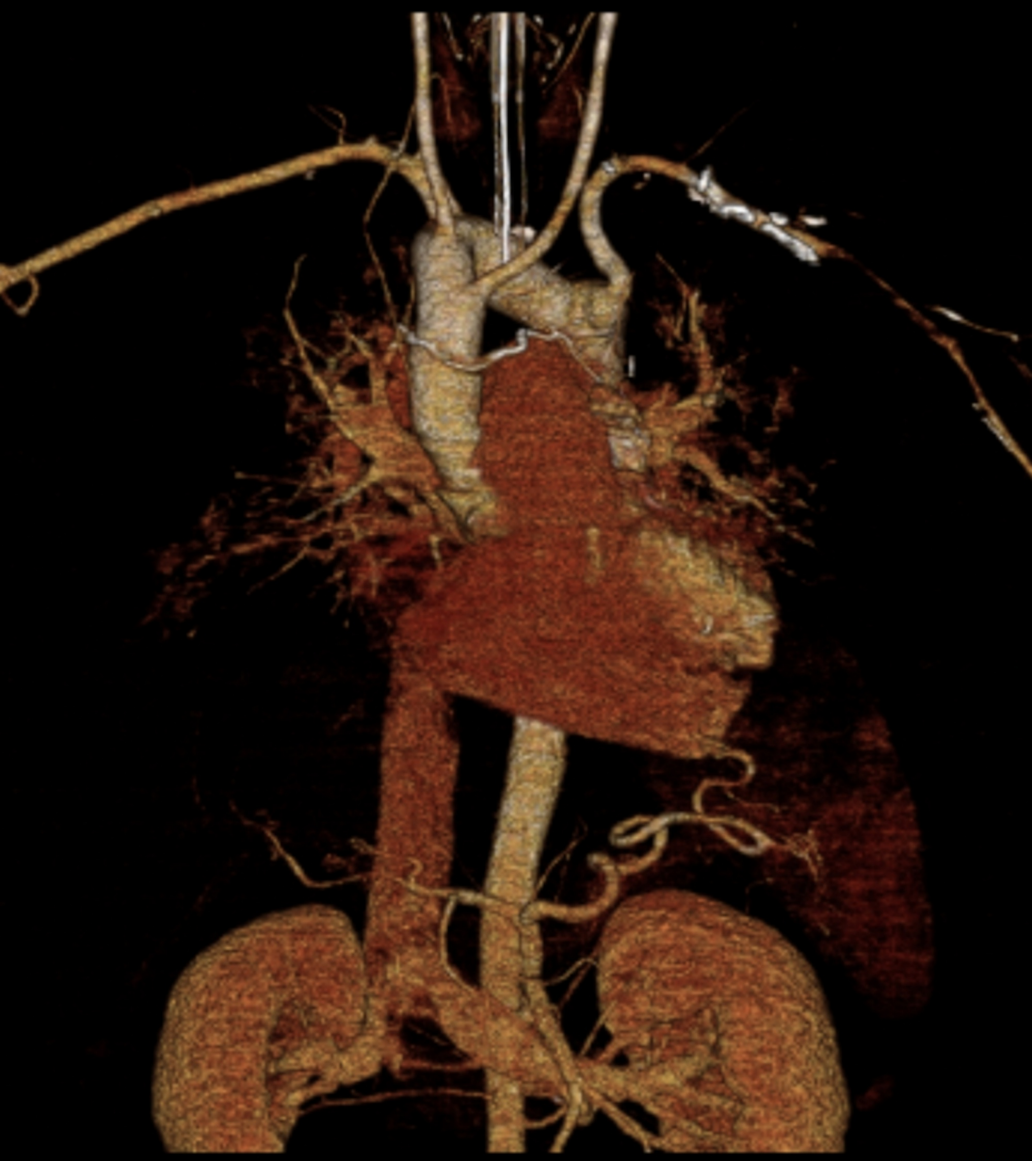
Three-dimensional volume-rendered image of patient’s right aortic arch, aberrant brachiocephalic artery, retro-esophageal left subclavian artery, and diverticulum of Kommerell. Given the patient’s tortuous vascular anatomy, access to the left common carotid artery (LCCA) via the femoral artery proved unfavorable; radial access was also ruled out and a direct left common carotid artery puncture was performed to gain rapid intracranial access.

A 36-year-old male with benign hypertension but otherwise no significant previous medical or surgical history presented to the emergency department with left gaze preference and right-side hemiparesis after being found down in his home with unclear time of onset of symptoms. CT angiography (CTA) revealed a distal left M1 middle cerebral artery (MCA) occlusion with an Alberta stroke program (ASPECTS) score of 9 on non-contrast CT of the head. Based on a favorable ASPECTS score, plans were made to proceed with endovascular mechanical embolectomy and the patient was transported to the neurointervention suite. Access to the right common femoral artery was obtained, and subsequent efforts were made to catheterize the left common carotid artery (LCCA). Initial attempts were unsuccessful at catheterizing the LCCA, and subsequent arch aortogram revealed that the patient had an aortic arch anatomy consistent with a congenital vascular rare variant of the great vessels. Volume-rendered 3D images from a concurrent CTA chest examination revealed a right aortic arch with the LCCA arising from the ascending aorta, as well as aberrant course of the brachiocephalic artery (Figure 1). Several consecutive attempts to catheterize the LCCA were unsuccessful due to unfavorable trajectories off the aortic arch, at which point it was decided to proceed with an alternative access route. Given the aforementioned anatomical variant, it was unlikely that a transradial approach to the LCCA would be successful; thus, it was decided to perform a direct left common carotid artery puncture to gain intracranial access.

**Figure 2:**
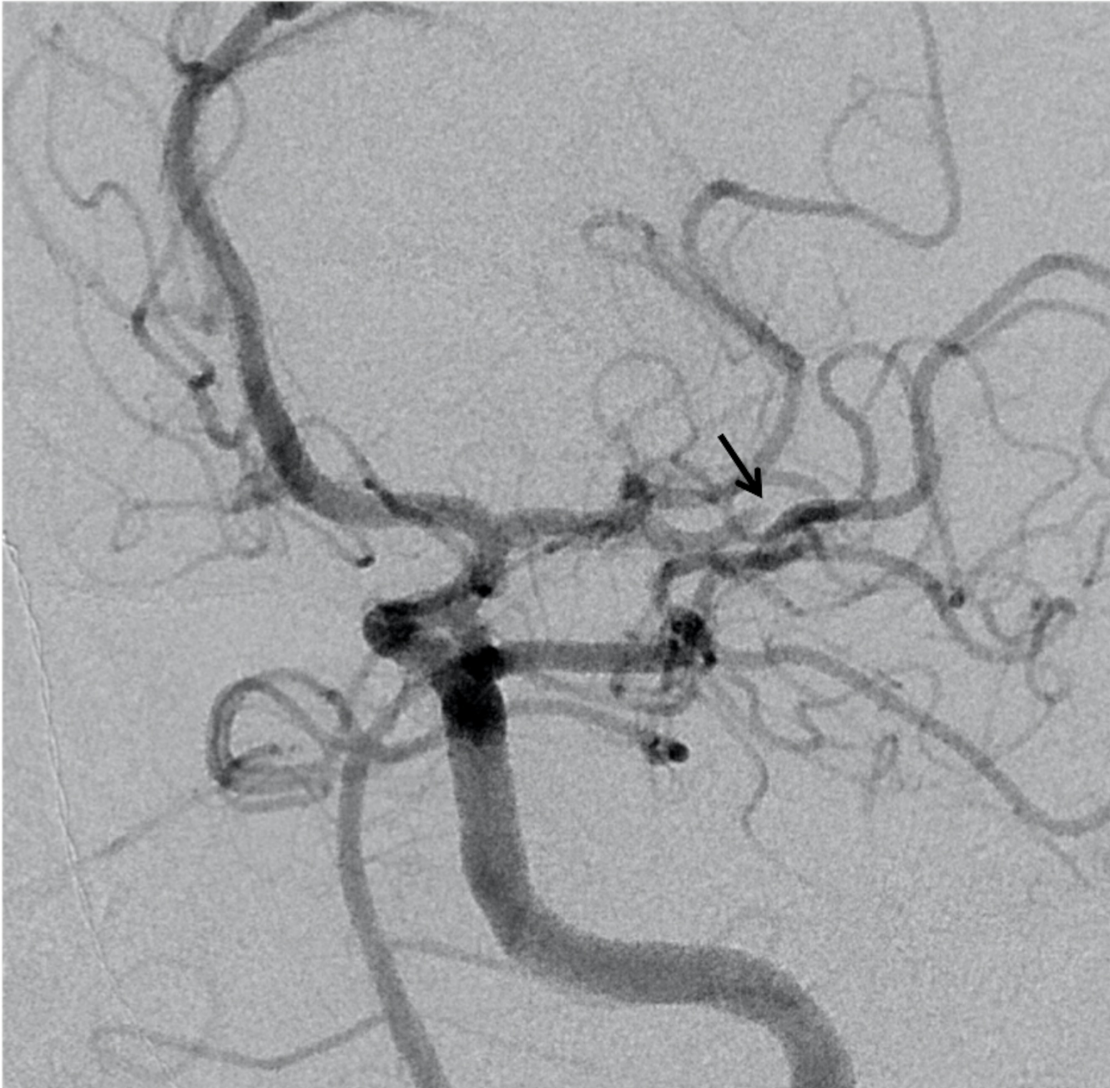
Pre-embolectomy diagnostic cerebral angiogram. Cerebral angiography revealed interval autolysis of the clot and recanalization of the distal left M1 middle cerebral artery (MCA) occlusion.  There is however persistent occlusion of an M2 division (black arrow) supplying an eloquent MCA territory.

**Figure 3:**
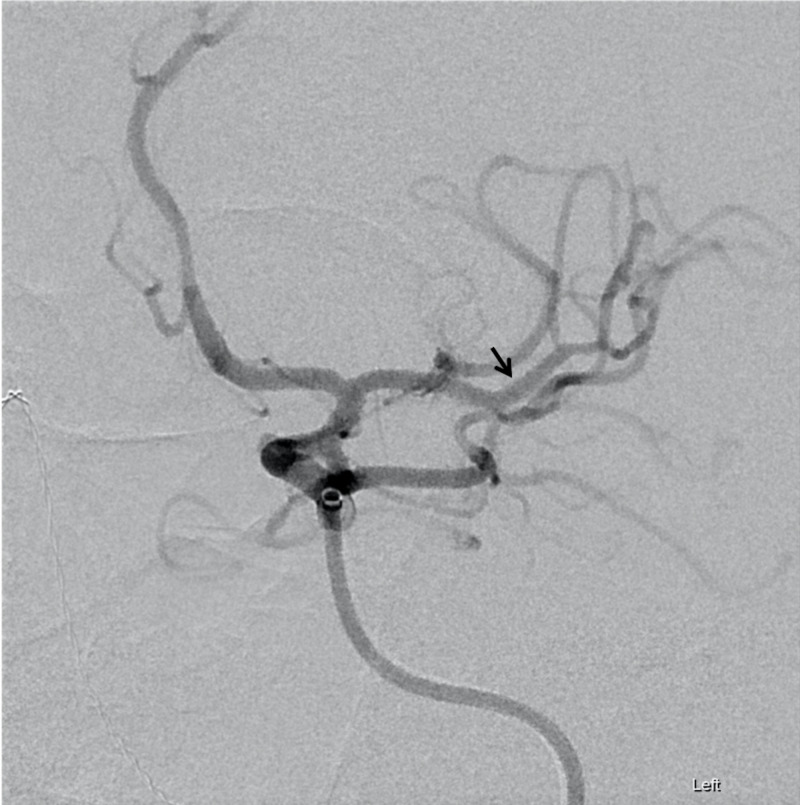
Post-embolectomy cerebral angiogram. Endovascular mechanical embolectomy was performed using a 4 mm x 20 mm Solitaire stentreiver device in conjunction with aspiration with Penumbra ACE68 catheter. Post-embolectomy cerebral angiography demonstrates recanalization of the previously-occluded middle cerebral artery (MCA) branch (black arrow).

The left neck was prepared and draped, and a suitable puncture site was confirmed. After administration of 1% lidocaine, ultrasound-guided access to the left common carotid artery was gained (Figure 2). A 6-French 10-cm sheath was placed at this time. After ensuring proper placement in the left MCA, endovascular mechanical embolectomy was performed with a 4 mm x 20 mm Solitaire stentreiver device (Medtronic Inc, Minneapolis, MN, USA) in conjunction with aspiration with Penumbra ACE68 catheter (Penumbra Inc, Alemeda, CA, USA) (Figure 3). At the conclusion of the procedure, the LCCA arteriotomy site with closed by the vascular surgery team via open surgical repair. The patient subsequently recovered remarkably well at post-procedure follow-up with a three-month modified Rankin score of 1. 

## Discussion

It is well-established that ischemic stroke leads to rapid destruction of surrounding brain parenchyma [3]. Time spent performing recanalization during stroke intervention is a crucial indicator of patient outcomes; thus, rapid diagnosis and treatment remain integral components in management of patients suffering from acute ischemic stroke [4]. Patients with unique aortic arch anatomy present challenges to accessing the site of occlusion using standard transfemoral or transbrachial/transradial approaches, which may significantly prolong the duration of the procedure. In our case, traversal of the aortic arch proved unsuccessful via the most common transarterial approach; thus, the left common carotid artery was chosen as the most feasible access site. Despite somewhat increased risk of hematoma formation and arterial dissection [5], direct carotid artery puncture may circumvent problematic or untraversable anatomy, crucially shortening the time from access to recanalization for this otherwise devastating aliment. 

## Conclusions

Direct common carotid artery puncture is a feasible alternative method of access for rapid intervention of acute embolic stroke in individuals with tortuous or otherwise difficult-to-navigate aortic arch anatomy. In patients with acute embolic stroke with a known rare variant of the aortic arch, treatment time may be further reduced by opting for direct common carotid artery puncture as the primary site of vascular access, though some patients with aberrant arch anatomy may remain asymptomatic and undiagnosed until the time of intervention.

## References

[1] Hanneman K, Newman B, Chan F (2017). Congenital variants and anomalies of the aortic arch. Radiographics.

[2] Faggioli GL, Ferri M, Freyrie A (2007). Aortic arch anomalies are associated with increased risk of neurological events in carotid stent procedures. Eur J Vasc Endovasc Surg.

[3] Saver JL (2006). Time is brain—quantified. Stroke.

[4] Hassan AE, Chaudhry SA, Miley JT (2013). Microcatheter to recanalization (procedure time) predicts outcomes in endovascular treatment in patients with acute ischemic stroke: when do we stop?. Am J Neuroradiol.

[5] Mokin M, Snyder KV, Levy EI (2015). Direct carotid artery puncture access for endovascular treatment of acute ischaemic stroke: technical aspects, advantages and limitations. J Neurointerv Surg.

